# Neural network dynamics underlying the bottom-up perception and top-down regulation processes of empathy

**DOI:** 10.1162/IMAG.a.972

**Published:** 2025-11-03

**Authors:** Xiao Wu, Ya-Zhuo Kong, Li Hu

**Affiliations:** State Key Laboratory of Cognitive Science and Mental Health, Institute of Psychology, Chinese Academy of Sciences, Beijing, China; Department of Psychology, University of Chinese Academy of Sciences, Beijing, China

**Keywords:** empathy, bottom-up perception process, top-down regulation process, dynamic causal modeling, Bayesian model selection

## Abstract

Contemporary theoretical models of empathy propose that it involves both bottom-up perception and top-down regulation processes. Although previous studies have explored brain regions involved in empathy, direct evidence for a hierarchical organization in empathy processing remains limited. In this functional magnetic resonance imaging study, we sought to address this gap by using positive, negative, and neutral stimuli to explore whether shared or valence-specific processing underlies positive and negative empathy. Our results indicate that the bottom-up perception process involves the bilateral triangular part of the inferior frontal gyrus (IFGtri) and the superior temporal sulcus (STS), with the IFGtri initiating information flow to the STS. Moreover, while bilateral IFGtri only shows excitatory effects on bilateral STS, bilateral STS only shows inhibitory effects on bilateral IFGtri. The right IFGtri appears to be more involved in external information processing, whereas the left IFGtri focuses more on internal information communication. The top-down regulation process involves information flow from the left dorsolateral superior frontal gyrus (dlSFG) to the medial prefrontal gyrus (mPFC), and then to the precuneus. Moreover, positive stimuli show an inhibitory effect from dlSFG to mPFC and from mPFC to precuneus, whereas negative stimuli only inhibit the flow from mPFC to precuneus. This finding suggests that more regulatory neural mechanisms occur to others’ positive emotions than negative ones. In conclusion, our study provides direct evidence of a hierarchical neural network supporting theoretical models of empathy, offering novel insights into how bottom-up perception and top-down regulation processes are instantiated in the brain.

## 1 Introduction

Empathy reflects one’s ability to perceive, understand, share, and respond to the emotional states of others (Decety & Jackson, 2006; Lulla et al., 2024; Riess, 2017; Wu et al., 2024; Yavuz et al., 2024). Depending on the affective valence of others’ emotion, empathy can be categorized as either positive or negative (Morelli et al., 2015; Wu et al., 2025). Over the past few decades, empathy models have been continuously evolving, and some important theoretical models have been proposed to describe the processes underlying empathy. In 2002, Preston and de Waal introduced the perception–action model (PAM), which posits that perceiving others’ emotions activates corresponding brain regions in the observer, subsequently triggering somatic and autonomic responses (Preston & de Waal, 2002). Specifically, they proposed that certain brain regions involved in motor observation and motor imagery may contribute to the perception process, including the left inferior frontal gyrus (IFG), right superior parietal lobule, superior temporal sulcus (STS), inferior parietal lobule, rostral supplementary motor area, left premotor cortex, middle frontal cortex, and cerebellum. Although the PAM primarily focuses on the perception–action process of empathy that occurs in different species such as chimpanzees and humans, it also mentioned the existence of cognitive forms of empathy as a result of prefrontal functioning. Later, in 2008, Decety and Meyer proposed a social-developmental neuroscience model of empathy that integrates three major components: (i) automatic affective sharing via perception–action coupling, (ii) self–other distinction, related to the temporoparietal junction (TPJ), and agency attribution, and (iii) executive functions, implemented in the prefrontal cortex (PFC), for regulation and control (Decety & Meyer, 2008). These components are embedded in a dynamic interplay between bottom-up resonance and top-down modulation, which jointly determine whether empathic responses lead to self-oriented personal distress or to other-oriented empathic concern or both. Specific and interacting neural circuits, including aspects of the PFC, insula, limbic system, and frontoparietal networks, are involved in the empathy process. In 2018, Heyes proposed a dual system model of empathy, in which Empathy 1 (emotional contagion) and Empathy 2 (empathic understanding) could solely or jointly represent an empathic response (Heyes, 2018). In Empathy 1, the emotional stimulus automatically triggers a motoric and/or somatic response via neural circuits in areas including the premotor cortex, inferior parietal lobe, and posterior STS (motor activation), and the anterior insula and anterior cingulate cortex (somatic activation). The controlled processing of Empathy 2 may involve metacognitive as well as cognitive appraisal. Although these models emphasize somewhat different processes (e.g., self–other awareness, metacognitive), they all agree that three core processes are involved: a bottom-up perception process, an automatic affective response, and a top-down regulation process. Specifically, an automatic perception triggers an immediate emotional response, which then becomes the input for a controlled cognitive process (Decety & Meyer, 2008; Heyes, 2018; Preston & de Waal, 2002).

Furthermore, in experimental psychology and medicine, five distinct subcomponents of empathy were mentioned: emotion recognition (the ability to identify others’ emotional states), affective empathy (emotional responses elicited by others’ experiences, e.g., personal distress), motivational empathy (the intrinsic altruistic drive motivating empathic behaviors, e.g., empathic concern), behavioral empathy (manifestations of empathic actions, such as helping, comforting, or reduced discrimination), and perspective taking (the capacity to adopt others’ viewpoints), with emotion recognition and affective empathy as the core concepts (Decety & Jackson, 2004; Mercer & Reynolds, 2002; Weisz & Zaki, 2018; Wu et al., 2024; Zaki & Ochsner, 2012). Additionally, theory of mind—the ability to understand others’ mental states, including beliefs and intentions—is consistently implicated in empathic processes (Byom & Mutlu, 2013; Decety & Meyer, 2008; Edmonds et al., 2024). Various influential factors distinctly or jointly impact these empathy sub-components. These factors generally fall into three main categories: characteristics of the subject (the individual experiencing empathy), features of the object (the individual being empathized with), and the nature of their relationship (Wu et al., 2024). Integrating the multi-componential nature of empathy with the above theoretical models suggests that bottom-up perception processes primarily correspond to emotion recognition, automatic affective responses align with affective empathy, and top-down regulation processes reflect the modulation by diverse influential factors. More specifically, emotion recognition can be regarded as the initial input stage of empathy, in which others’ emotions are perceived and represented within oneself (bottom-up process). In contrast, regulatory processes integrate and balance various influential factors to modulate downstream empathic subprocesses (top-down modulation).

Existing neuroimaging studies have provided substantial evidence regarding the neural mechanisms of empathy. For example, bilateral IFG and left STS exhibit stronger responses during emotion recognition than gender identification (Voorthuis et al., 2014). Brain regions typically associated with theory of mind, such as the medial PFC (mPFC) and the precuneus/posterior cingulate cortex, have been implicated in both positive and negative empathy (Morelli et al., 2014; Perry et al., 2012; Wu et al., 2025). However, although these studies identify relevant brain regions, they do not provide direct evidence for a hierarchical neural organization of empathy processing. For instance, while the IFG and STS are known to be involved in emotion recognition, the way in which these regions interact during empathic processing remains unclear. In other words, direct neural network-level evidence that supports theoretical models of empathy is still lacking. Moreover, most existing models pay little attention to the distinction between positive and negative empathy. Among the three core processes, their primary difference lies in the automatic affective responses (e.g., smiling in positive contexts vs. frowning in negative contexts). Nevertheless, both positive and negative empathy likely share fundamental elements of bottom-up perception (e.g., recognizing emotional expressions) and top-down regulation (e.g., considering contextual and relational factors).

Given the complexity of empathy, it is unrealistic to fully delineate how all empathic processes operate as an integrated neural network in a single study. Therefore, the present work aims to examine the bottom-up perception and top-down regulation processes, and to identify shared as well as valence-specific mechanisms underlying positive and negative empathy, as an initial step toward uncovering the hierarchical neural architecture of empathy. Specifically, we conducted a functional magnetic resonance imaging (fMRI) study using positive, negative, and neutral stimuli to evoke positive and negative empathic responses. Each stimulus featured a high-resolution image showing facial expressions and concise text describing real-life events from online news and documentary films. By applying general linear model (GLM) analyses to the fMRI data, we identified brain regions involved in bottom-up perception and top-down regulation processes. Then, we applied dynamic causal modeling (DCM) and Bayesian model selection to examine how information flows within the brain regions responsible for both processes (Chen et al., 2024; Di Cesare et al., 2024; Song et al., 2021; Zhao et al., 2021). DCM is a hypothesis-driven approach for investigating distributed neural architectures underlying observed brain responses, and it is particularly well suited for fMRI data (Friston, 2011). Since we aimed to test a hypothesis on the neural dynamics of empathy process, this approach offers a direct means of examining whether and how a hierarchical neural network underlies empathy processing.

## 2 Methods

### 2.1 Participants

As we primarily contrasted two types of within-subject stimuli, we performed a priori power analysis for paired-sample t-tests using G*power (Faul et al., 2007, 2009). The analysis was set at a statistical power of 0.8, with an effect size of 0.5 and a significant level of 0.05 (two-tailed). Results indicated a minimum sample size of 34 was required to detect the main effects between 2 dependent means. To account for 1 participant who provided incomplete data due to excessive head motion (>2.4 mm) during the final 3 min of a run, leading to the removal of 10 trials (7 negative and 3 neutral), a total of 35 healthy, right-handed participants were ultimately recruited (mean age = 22.03 ± 2.30 years; 17 males). This sample size is considered sufficient for a within-subject design involving DCM analysis according to recent similar publications (Di Cesare et al., 2024; Liu et al., 2025; Zhao et al., 2021). Age and years of education did not differ significantly between male and female participants (*p* > .05). The study was approved by the ethics committee of the Institute of Psychology, Chinese Academy of Sciences, China, and all participants provided written informed consent.

### 2.2 Experimental design

**Fig. 1.**
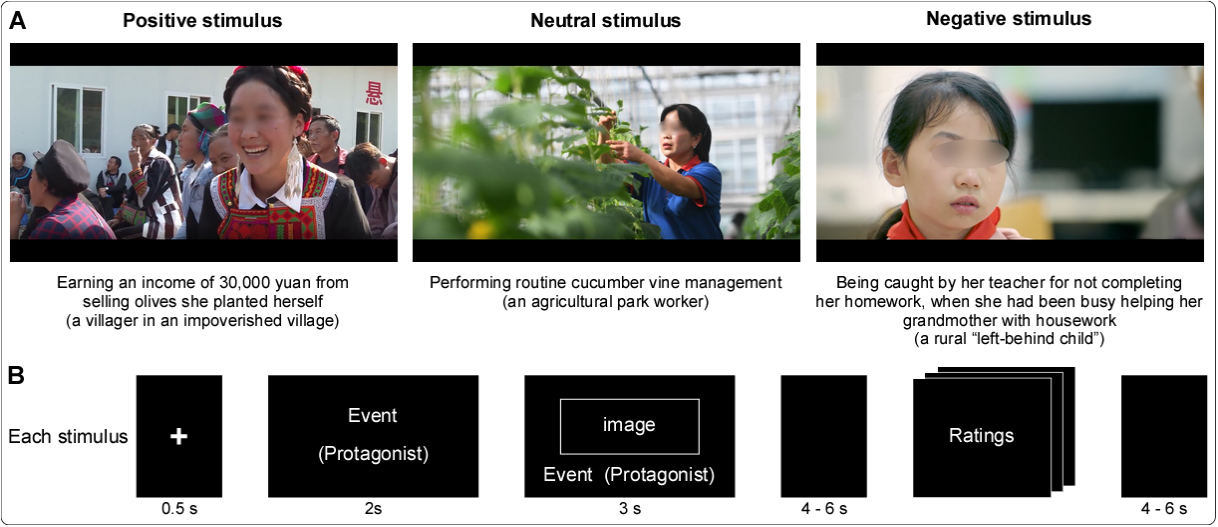
Representative stimuli and experimental design. (A) Examples of positive, neutral, and negative stimuli, each with an image showcasing the corresponding facial expressions, as well as text describing both the event and the protagonist. (B) Each participant completed 4 runs, each comprising 24 stimuli (16 positive/negative and 8 neutral stimuli). A stimulus sequence began with a 0.5-s white cross, followed by a 2-s text screen and a 3-s image screen. After a 4- to 6-s jittered interval, participants were prompted to identify the protagonist’s emotion, rate their own evoked emotion and motivational empathy.

A total of 96 stimuli were presented with an equal distribution across 3 emotional valences: positive, negative, and neutral (Fig. 1A). Positive stimuli were uplifting events with happy facial expressions, for example, a villager in an impoverished village earning an income of 30,000 yuan from selling olives she planted herself. Negative stimuli involved distressing events with sad facial expressions, for example, a rural “left-behind child” being caught by her teacher for not completing her homework, when she had been busy helping her grandmother with housework. Neutral stimuli depicted everyday occurrences with neutral facial expressions, for example, an agricultural park worker performing routine cucumber vine management. Detailed stimulus information is available at https://osf.io/npbrg. Note that the final set of 96 stimuli was selected from an initial pool of 120 based on evaluations conducted by an independent sample (n = 30; 15 males; mean age = 23.27 ± 3.52 years). All protagonists depicted in the stimuli were ordinary individuals—not celebrities or public figures—and were matched across the three valence conditions (positive, neutral, and negative) in terms of age group, nationality, and gender. The length of Chinese characters used to describe both the protagonist and the event (which was shorter than the English translation) was also balanced across conditions (all *p*s > .05). Affective valence ratings were as follows: positive (2.54 ± 0.29), negative (-2.87 ± 0.46), and neutral (0.25 ± 0.35). Arousal ratings were positive (4.62 ± 0.52), negative (3.97 ± 0.55), and neutral (2.30 ± 0.47). To examine whether stimulus attributes influenced participants’ evaluations, a linear mixed effects models was built, with protagonist’s sex, age group, and nationality as fixed effects, and participant and stimulus as random intercepts. No significant effects were found for any of these variables (all *p*s > 0.05).

The experimental procedure consisted of 4 runs: 2 for positive stimuli and 2 for negative stimuli (16 positive/negative and 8 neutral stimuli in each run; Fig. 1B). The order of positive and negative runs was counterbalanced separately within male and female participants. This approach was intended to prevent any systematic bias or emotion carryover effects that could arise if, for example, most male participants experienced the positive condition first while most female participants began with the negative condition. A resting-state fMRI scan (~7 min) was inserted between the two positive and negative runs to minimize carryover emotional effects. Each stimulus consisted of a 0.5-s white cross, followed by a 2-s text screen (describing the event and protagonist in the upper and lower segments, respectively) and a 3-s image screen (showing the protagonist’s facial expression with text information at the bottom in a small font). After each stimulus, participants were prompted to rate 2 questions (question #1: What is the emotional state of the protagonist? question #2: What emotion do you feel for them?) on a 9-point Likert scale ranging from -4 (very sad) to 4 (very happy), capturing the perceived emotions of the protagonist and the participant’s own emotion evoked for the protagonist. Participants were also required to rate an additional question on their own motivational empathy (question #3: To what extent, do you feel warmth, kindness, concern, or compassion?) ranging from 1 (very little) to 9 (very much). Please note that only the first two ratings were used in this study. Jittered inter-stimulus intervals ranging from 4 to 6 s were implemented between subsequent trials and between the image screen and the rating period (Thompson et al., 2014).

### 2.3 Behavioral data analysis

Ratings on the protagonists’ emotions and participants’ own emotions were compared using one-way repeated-measures analyses of variance (ANOVAs). Multilevel correlation analyses were conducted to examine how strongly the protagonists’ emotions influenced participants’ own emotions in each condition. The Python *pingouin* package (Vallat, 2018) was used for ANOVA, and the R *correlation* package (Makowski et al., 2020, 2022) was used for multilevel correlation analyses. A false discovery rate (FDR) correlation was applied to control for multiple comparisons.

### 2.4 MRI data acquisition and preprocessing

All MRI data were acquired using a GE Discovery MR 750 3.0T scanner with a 32-channel head coil at the Magnetic Resonance Imaging Research Center, Institute of Psychology, Chinese Academy of Sciences, China. The MRI acquisition parameters and preprocessing steps are detailed in the Supplementary Materials (“MRI Acquisition and Preprocessing”). The preprocessing was conducted using ANTS 2.5.1 (Tustison et al., 2010) and the SPM12 toolbox (Statistical Parametric Mapping, http://www.fil.ion.ucl.ac.uk/spm). After preprocessing, the four fMRI runs were concatenated following SPM guidelines, as required for subsequent DCM analyses.

### 2.5 Regions of Interest

Unlike well-established domains such as sensory (e.g., visual (Di Cesare et al., 2024; Park et al., 2019) and auditory (Bai et al., 2025; Sarmukadam et al., 2025)) and motor processing (Yang et al., 2023; Zou et al., 2024), the neural substrates of empathy—particularly its subcomponents (e.g., emotion recognition, affective empathy, motivational empathy, and perspective taking)—are less clearly defined and lack universally accepted solutions. Given this limitation, our initial step necessarily involved identifying candidate ROIs based on converging evidence from our fMRI activation patterns, complemented by the existing literature.

GLM analyses were performed to determine the regions of interest (ROIs) for the following DCM analyses. At the first level, three regressors (positive, neutral, negative) were included in GLM, and the corresponding *t* contrasts for each regressor were estimated. Six head motion parameters were included as regressors of no interest. The image screen was used as the trial onset, since brain responses to facial expressions are faster and more direct than those to words (Schacht & Sommer, 2009).

Two series of second-level analyses were then performed. The first aimed to identify brain regions activated across all three conditions. Specifically, one-sample t-tests were performed for each of the three conditions. Brain regions that were jointly activated in all three conditions (through *ImCalc* in SPM to perform a conjunction analysis) and have been shown to correlate with recognizing others’ emotions were identified as the starting point of automatic bottom-up perception process. Age, sex, and stimulus order (positive runs first or negative runs first) were included as covariates. The second series sought to determine brain regions that showed stronger activations to both positive and negative stimuli than neutral stimuli. Specifically, a within-subject one-way repeated-measures ANOVA was performed at the group level, which was followed by a conjunction analysis of two *t* contrasts (Positive > Neutral and Negative > Neutral). These brain regions, showing stronger activations for both positive and negative empathy, may be involved in a later stage of perception process and the shared top-down regulation process of both positive and negative empathy. A voxel-level threshold of *p* < .001 and a cluster-level family-wise error (FWE)-corrected threshold of *p* < .05 were applied to account for multiple comparisons.

### 2.6 First-level DCM analyses

DCM is a generic Bayesian framework for inferring hidden neuronal states from measured brain activity. In DCM, three matrices, that is, matrices A, B, and C, are built to describe the network structure and experimental influences. Matrix A represents the fixed intrinsic self-connections within each brain region and extrinsic connections between brain regions. Matrix B represents the changes in fixed connections produced by stimuli. Matrix C represents the rate of change of the neural response due to the driving inputs. It is acknowledged that driving inputs (matrix C) usually represent brief external events that “ping” specific regions in the neural network at the onset of each stimulus (Friston, 2011), with subsequent effects propagating through the network via intrinsic (matrix A) and modulatory (matrix B) connections. By estimating these matrices, DCM addresses the following questions (Friston et al., 2003; Hartwigsen et al., 2015; Stephan et al., 2010): (1) Is brain region A responsible for activation changes in brain region B? (2) Which connections are modulated by experimental stimuli? (3) Where does the sensory input(s) enter the network? Furthermore, Bayesian model comparison can be employed to determine the best fitting hypothesis about the underlying mechanisms from a set of competing models (Stephan et al., 2009).

Since the automatic bottom-up perception and top-down regulation processes were theoretically distinct, we built two separate DCMs to capture their respective information flow. DCM#1 was designed to detect the perception process that differentiates emotional from neutral facial expressions, while DCM#2 aimed to detect the shared regulation process between positive and negative empathy. Note that we did not differentiate between positive and negative stimuli in DCM#1, because emotion recognition showed a shared encoding scheme for both positive and negative facial expressions (see Section 3.2 for details). Two key ingredients are required for both DCMs: an ROI time series and a design matrix. Consequently, we created a separate GLM for each DCM—GLM#1 for DCM#1 and GLM#2 for DCM#2. In GLM#1, the first regressor (“Face”) encompassed all three conditions (i.e., positive, neutral, and negative) and the second regressor (“Emotion”) included only positive and negative stimuli. In GLM#2, three regressors (one for each condition) were included. Accordant *t* contrast for each regressor was built at the first level with an additional “Emo-Neu” contrast representing Positive & Negative > Neutral in GLM#2. To determine the group-level peak coordinates for brain regions involved in the initial perception process, one-sample t-tests were performed for the “Face” contrast in the second-level GLM#1. The peak coordinates for other ROIs were determined via a conjunction analysis of two *t* contrasts (Positive > Neutral and Negative > Neutral) in the second-level GLM#2. During time series extraction, each ROI’s peak coordinates were constrained to lie within 10 mm of the group-level GLM peak, and each ROI was defined as the cluster of voxels within a 5-mm sphere centered on its peak coordinates from the first-level GLM analysis (the “Face” contrast in GLM#1 for regions initiating the perception process; the “Emotion” contrast in GLM#1 for regions in the later perception process; the “Emo-Neu” contrast in GLM#2 for regions involved in the regulation process). These approaches were conducted to accommodate individual anatomical and functional variations. A voxel-level threshold of *p* < .001 was initially applied at the first-level GLM to exclude the noise voxels in ROIs. If no voxels surpassed this threshold, it was incrementally relaxed to *p* < .01 and then *p* < .05 (Zeidman et al., 2019a). Since we aimed to explore commonalities rather than individual differences, participants without any above-threshold voxels (*p* < .05) were excluded from the DCM analyses. Then, the first eigenvariate of the blood-oxygenation-level-dependent (BOLD) signal in each ROI was extracted after mean correction by an *F* contrast capturing the main effect of all regressors of interest (“Face” and “Emotion” in GLM#1; “Positive”, “Neutral”, and “Negative” in GLM#2).

The model design matrices for DCM#1 (perception process) and DCM#2 (regulation process) were tailored to their respective conceptual requirements. In DCM#1, the task input (matrix C) was predetermined, and the primary focus was on variations in the modulatory matrix B. By contrast, in DCM#2, top-down regulation was modeled as the modulatory effect (matrix B), prompting a shift in focus to matrix C to identify which ROI(s) served as the driving input for the stimuli. To determine the most appropriate model, multiple reduced models were built for each DCM, each including only parts of its respective target matrix.

### 2.7 Second-level DCM analyses with a Parametric Empirical Bayes model

After estimating each participant’s full model, we built a group-level Parametric Empirical Bayes (PEB) model that incorporated the connectivity parameters of interest from all participants’ full models. The model quality is indexed by the free energy *F*, which approximates the log model evidence. At the group level, *F* is calculated as the sum of all participants’ DCM accuracies minus the complexity introduced by fitting individual DCMs and the second-level GLM. To estimate the free energy and parameters of reduced models, we employed a procedure referred to as Bayesian Model Reduction (BMR), which treats a reduced model as the full model with a certain subset of connections switched off (i.e., fixed at zero). This method enables efficient evaluation of a large set of models in seconds. Then, we performed a Bayesian model comparison among the full and reduced models in a way conceptually analogous to an *F* test in classic statistics, yielding a posterior probability for each model relative to the one with the lowest evidence (Zeidman et al., 2019b). The model providing the best explanation for the data was identified.

We ran PEB analyses separately for DCM#1 and DCM#2. To summarize each parameter across all models and obtain numerical estimates, Bayesian Model Averaging (BMA) was computed. In BMA, parameter estimates are averaged over different models, weighted by each model’s posterior probability. To focus on parameters most likely to be nonzero, the BMA output was thresholded to retain only those parameters that had a 95% posterior probability of being present. Additionally, an automatic search was conducted to “prune” parameters that did not contribute to the model evidence. This procedure compares the evidence for reduced models using BMR, iteratively discarding parameters that do not contribute to model evidence. The iterative procedure stops when discarding any parameter starts to decrease model evidence. A BMA was then calculated over up to 256 models from the last iteration of the greedy search, yielding a final model that also takes uncertainty into account (Zeidman et al., 2019b). We report all parameters with a posterior probability above .95 of being present. Mean-centered age, sex, and the stimulus order (positive runs first or negative runs first) were included as covariates of no interest.

To present the full connectivity matrices for both perception (DCM#1) and regulation (DCM#2) processes, we specified PEB models for the other matrices (matrices A and C for DCM#1, matrices A and B for DCM#2). An automatic search over models was also performed to prune parameters that did not contribute to model evidence.

## 3 Results

### 3.1 Behavioral results

Significant differences were observed in the perceived emotions of the protagonists among three conditions, *F*(2,68) = 1353.76, *q*_FDR_ < .001; positive: 3.29 ± 0.94, 95% confidence interval (CI) [3.24, 3.35]; neutral: 0.56 ± 1.10, 95% CI [0.49, 0.62]; negative: -3.40 ± 0.88, 95% CI [-3.45, -3.34], as well as in participants’ own emotions for the protagonists’ experiences, *F*(2,68) = 370.44, *q*_FDR_ < .001; positive: 2.48 ± 1.26, 95% CI [2.41, 2.56]; neutral: 0.42 ± 0.99, 95% CI [0.36, 0.47]; negative: -2.75 ± 1.21, 95% CI [-2.82, -2.68]. Moreover, participants’ own emotions were significantly correlated with protagonists’ emotions (positive: *r* = .51, neutral: *r* = .68, negative: *r* = .69; all *q*_FDR_ < .001), indicating a successful experimental manipulation that evoked positive and negative empathy.

### 3.2 Regions of interest

GLM analysis showed shared significant activations across all three conditions in bilateral triangular part of the IFG (IFGtri), posterior orbitofrontal cortex (OFCpost), STS, the lingual gyrus extending to bilateral inferior occipital gyrus, and parahippocampal gyrus, as well as in the right hippocampal gyrus, and right amygdala (Supplementary Table S1). Shared enhanced activations for both Positive > Neutral and Negative > Neutral contrasts were found in bilateral STS, the left dorsolateral superior frontal gyrus (dlSFG), the mPFC, and the precuneus (Supplementary Table S2).

**Fig. 2.**
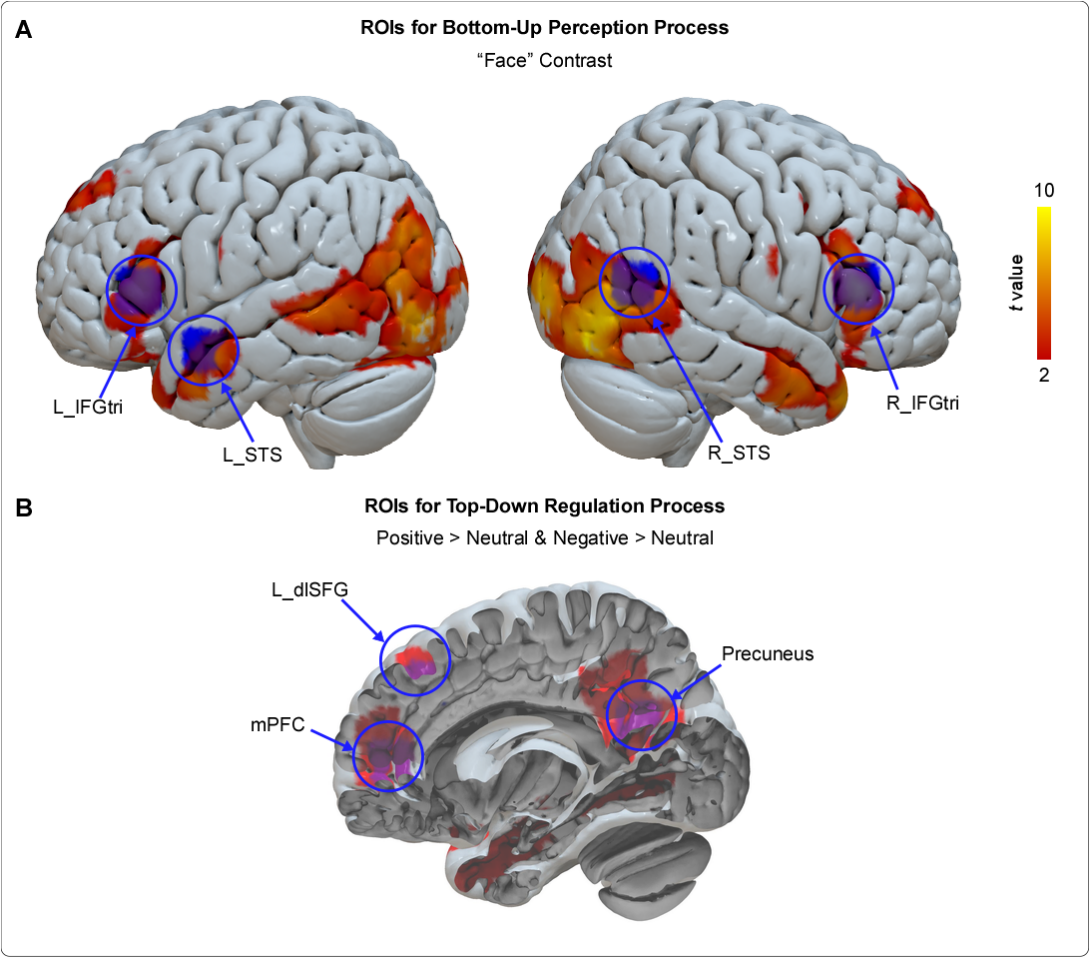
Regions of interest to model the bottom-up perception process (A) and top-down regulation process (B) in dynamic causal modeling analyses. ROIs are depicted in blue and circled for emphasis. (A) Brain activations across all three conditions (i.e., positive, neutral, and negative) are shown using a red-to-yellow color scale. (B) Conjunction results for Positive > Neutral and Negative > Neutral contrasts are highlighted in red. L: Left, R: right; IFGtri: triangular part of the inferior frontal gyrus; STS: superior temporal sulcus; dlSFG: dorsolateral superior frontal gyrus; mPFC: medial prefrontal cortex.

Ultimately, bilateral IFGtri (left: [-56, 30, 10]; right: [54, 30, 16]) was identified as the ROIs reflecting the starting region of the perception process for the following reasons: (1) The IFG is theorized to be involved in the perception process within the PAM, a hypothesis supported by its activation during motor observation tasks (Preston & de Waal, 2002), (2) bilateral IFGtri showed robust activations across all three conditions (right IFGtri: 883 voxels, *t* = 9.87; left IFGtri: 536 voxels, *t* = 6.70), and (3) bilateral IFGtri demonstrated negative correlations with protagonists’ emotions (i.e., ratings for question #1) in our study for both positive (right IFGtri: 228 voxels, *t* = 5.24; left IFGtri cluster1: 49 voxels, *t* = 4.97; left IFGtri cluster2: 63 voxels, *t* = 4.43) and negative (right IFGtri: 49 voxels, *t* = 4.52; left IFGtri: 86 voxels, *t* = 5.24) stimuli in parametric modulation analyses that can identify brain regions whose activations covary with task-related parameters; and it showed weaker response in emotional stimuli than in neutral stimuli (see more details in Wu et al., 2025). These observations underscore a close link between bilateral IFGtri and the neural processing of others’ emotions, supporting its role as an initial perceptual region. Bilateral STS (left: [-56, 2, -14]; right: [56, -62, 18]) was chosen as the ROIs reflecting the later stage of the perception process for the following reasons: (1) They showed enhanced activations for both Positive > Neutral and Negative > Neutral contrasts, (2) the STS is known to be a core region in face perception (Direito et al., 2019), (3) there is evidence of a direct fiber path between the STS and the IFG (Sarubbo et al., 2020; Suchan et al., 2014), and (4) both the IFG and the STS have been implicated as regions in the human mirror neuron system (Wallenwein et al., 2024). For the regulation process, three brain regions were defined as the ROIs: the left dlSFG: [-20, 32, 36], the mPFC: [-6, 48, 6], and the medial precuneus: [-2, -58, 22]. All these three ROIs showed enhanced activations for both Positive > Neutral and Negative > Neutral contrasts. The dlSFG, part of the dorsolateral PFC (DLPFC), has been extensively recognized as being critically involved in cognitive control, including emotion regulation (Rilling & Sanfey, 2009; Seminowicz & Moayedi, 2017). The mPFC is involved in understanding others’ psychological states (Mitchell et al., 2005) and has been proposed to serve as an integrative hub for emotional, sensory, social, memory, and self-referential processing (Gage & Baars, 2018). The precuneus plays a central role in episodic memory retrieval (Cavanna & Trimble, 2006) and is proposed to contribute to the formation of mental models by integrating current inputs with prior knowledge (Zysset et al., 2002). Notably, the mPFC and the precuneus, both key nodes of the default mode network and the theory of mind network (Carrington & Bailey, 2009; Spreng & Grady, 2010), are commonly linked to self-referential processing (He et al., 2025). Furthermore, previous studies have proven the causal interaction from the fronto-parietal central executive network (including the DLPFC) to the default mode network (including the mPFC and precuneus/posterior cingulate cortex) (Chen et al., 2013; Liston et al., 2014). In summary, bilateral IFGtri and bilateral STS were included as ROIs to model the bottom-up perception process (Fig. 2A), while the left dlSFG, the mPFC, and the precuneus were selected as ROIs to model the top-down regulation process in our DCM analyses (Fig. 2B).

### 3.3 DCM results

In DCM#1, all participants had at least one voxel exceeding the threshold (*p* < .05), allowing ROI time series to be extracted. However, in DCM#2, one participant had no above-threshold voxels, so no ROI time series could be obtained for that individual. This participant was excluded from DCM#2 analysis.

**Fig. 3.**
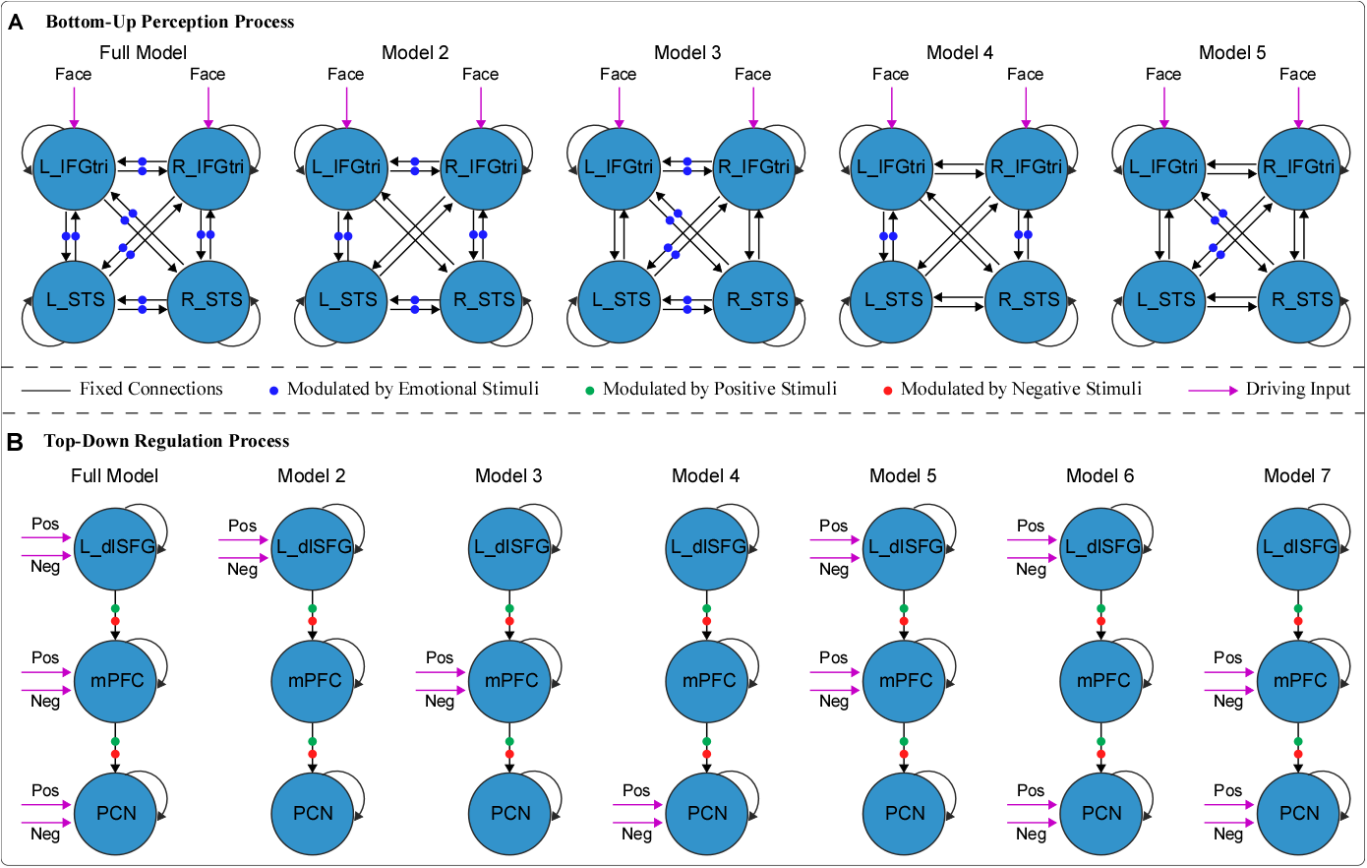
Full and reduced models for bottom-up perception (A) and top-down regulation (B) processes. Black lines indicate fixed intrinsic self-connections and extrinsic between-region connections (matrix A), and purple lines indicate the driving input of the process (matrix C). (A) Blue dots indicate modulatory effects of emotional stimuli (matrix B). (B) Green dots indicate the modulatory effects of positive stimuli, and red dots represent the effects of negative stimuli (matrix B). L: left, R: right; IFGtri: triangular part of the inferior frontal gyrus; STS: superior temporal sulcus; dlSFG: dorsolateral superior frontal gyrus; mPFC: medial prefrontal cortex; PCN: precuneus.

To build DCM#1, a full model was initially established (Fig. 3A, Full Model). Matrix A encompassed self-connections of all ROIs and all between-region connections among ROIs. Matrix B captured the modulatory effects of emotional stimuli (including both positive and negative stimuli) on the between-region connections. Matrix C represented the driving input of the perception process (i.e., bilateral IFGtri responses to all three types of stimuli). The corresponding reduced models featured a reduced matrix B (Fig. 3A, Models 2–5). For DCM#2, a full model was also established at first (Fig. 3B, Full Model). Matrix A included self-connections of all ROIs and top-down between-region connections. Matrix B reflected the modulatory effects of positive or negative stimuli on the top-down between-region connections. Matrix C indicated that all ROIs received driving inputs from either positive or negative stimuli. The corresponding reduced models featured a reduced matrix C (Fig. 3B, Models 2–7).

**Fig. 4.**
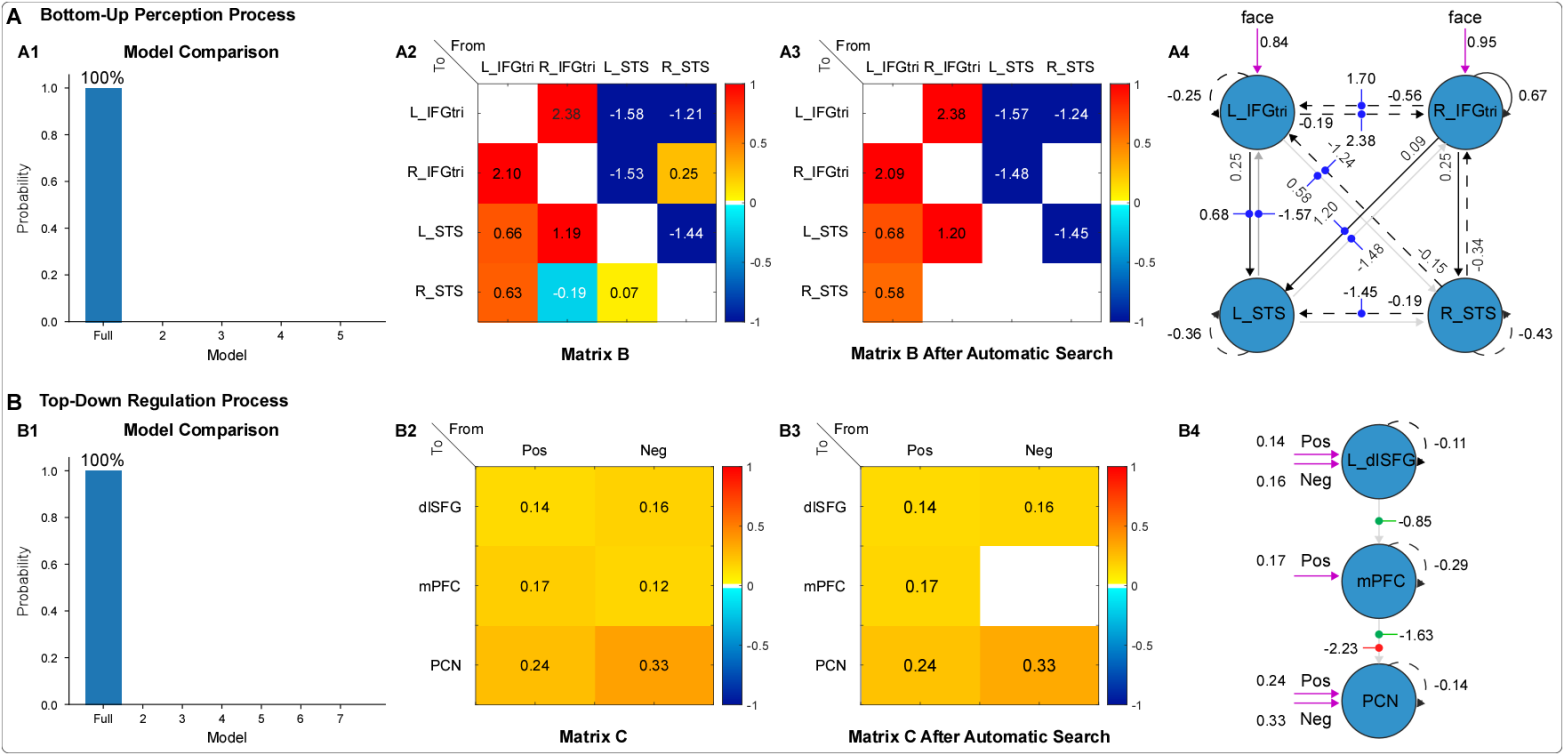
Model comparison results, effective connectivity matrices before and after automatic search, and full model matrices for perception (A) and regulation (B) processes. In Panels A2 and A3, positive signs (yellow and orange) indicate an increase in a fixed between-region connection (if it exists) or an excitatory effect (if it does not), whereas negative signs (turquoise and blue) indicate a decrease in a fixed between-region connection or an inhibitory effect. In Panels A4 and B4, black lines indicate significant fixed connections, with solid lines indicating excitatory effects and dotted lines indicating inhibitory effects; gray lines indicate not-significant fixed connections (matrix A). In Panel A4, blue dots indicate the modulatory effects of emotional stimuli (matrix B). In Panel B4, green dots indicate the modulatory effects of positive stimuli, and red dots represent the effects of negative stimuli (matrix B). Purple lines indicate the driving input (matrix C). L: left, R: right; Pos: positive; Neg: negative; IFGtri: triangular part of the inferior frontal gyrus; STS: superior temporal sulcus; dlSFG: dorsolateral superior frontal gyrus; mPFC: medial prefrontal cortex; PCN: precuneus.

For the perception process (DCM#1), PEB analysis showed that the full model provided the best fit to our data, with a posterior probability of 100% compared with plausible alternative reduced models (Fig. 4, A1). By thresholding the BMA of directed connectivity estimates at >95% posterior probability, we identified the connections (i.e., model parameters) modulated by emotional stimuli (Fig. 4, A2). Further exhaustive search of all possible reduced models revealed similar results but pruned away the modulatory effects on connections from the right IFGtri and left STS to the right STS, as well as from the right STS to the right IFGtri (Fig. 4, A3). In the final connectivity matrix after exhaustive search, positive modulations were observed only in between-region connections originating from bilateral IFGtri, and negative modulations were observed only in between-region connections originating from bilateral STS. In the context of the full matrices after exhaustive search (Fig. 4, A4), only excitatory effects (one region increases rate of change in another region) were observed on the between-region connections from bilateral IFGtri to bilateral STS, while only inhibitory effects (one region inhibits rate of change in another region) were observed on the between-region connections from bilateral STS to bilateral IFGtri, in both matrices A and B. For matrix A, bilateral IFGtri showed a fixed excitatory effect on the left (L) STS (L_IFGtri → L_STS: 0.25 Hz, R_IFGtri → L_STS: 0.09 Hz), and only the right (R) IFGtri excited the right STS (0.25 Hz). The right STS inhibited bilateral IFGtri (R_STS → L_IFGtri: -0.15 Hz, R_STS → R_IFGtri: -0.34 Hz), while the left STS had no fixed connection to bilateral IFGtri. For matrix B, in response to emotional stimuli, the left IFGtri showed increased excitatory effect on the left STS (0.68 Hz with 0.25 Hz in matrix A) and the right STS (0.58 Hz without fixed connection), and the right IFGtri showed increased excitatory effect on the left STS (1.20 Hz with 0.09 Hz in matrix A). Conversely, the left STS inhibited bilateral IFGtri (L_STS → L_IFGtri: -1.57 Hz, L_STS → R_IFGtri: -1.48 Hz, without fixed connection), and the right STS inhibited the left IFGtri (-1.24 Hz with -0.15 Hz in matrix A). Additionally, the right IFGtri showed a positive self-connection parameter (0.67), while all other regions showed negative self-connection parameters (L_IFGtri: -0.25, L_STS: -0.36, and R_STS: -0.43). These self-connection parameters are unitless log-scaling parameters that scale (multiply up or down) the default value of -0.5 Hz: A more positive value implies stronger self-inhibition (and thus a weak response to inputs from the network), while a more negative value implies weaker self-inhibition (Zeidman et al., 2019a). In summary, effective connectivity from bilateral IFGtri to bilateral STS was entirely excitatory in the fixed connections and excited or showed increased excitation in response to emotional stimuli. Conversely, effective connectivity from bilateral STS to bilateral IFGtri were entirely inhibitory in the fixed connections and inhibited or showed decreased inhibition in response to emotional stimuli. Notably, the right IFGtri was less affected by this perception network compared with the other three brain regions.

For the regulation process (DCM#2), a PEB analysis also showed that the full model best explained our data, with a posterior probability of 100% compared with reduced models (Fig. 4, B1). All three regions (i.e., the left dlSFG, the mPFC, and the precuneus) were identified as receiving driving inputs from both positive and negative stimuli (Fig. 4, B2). Further automatic search of reduced models showed similar results but pruned away mPFC from receiving driving inputs from negative stimuli (Fig. 4, B3). Specifically, positive stimuli generated an excitatory driving effect on the left dlSFG (0.14 Hz), the mPFC (0.17 Hz), and the precuneus (0.24 Hz), while negative stimuli generated an excitatory driving effect on the left dlSFG (0.16 Hz) and the precuneus (0.33 Hz). In the context of the full matrices after exhaustive search (Fig. 4, B4), no fixed between-region connections were observed among ROIs. In response to positive stimuli, inhibitory modulatory effects were observed in the between-region connections from the dlSFG to the mPFC (-0.85 Hz) and from the mPFC to the precuneus (-1.63 Hz). In response to negative stimuli, an inhibitory modulatory effect was observed only in the between-region connection from the mPFC to the precuneus (-2.23 Hz). These findings suggest distinct patterns of modulation depending on the emotional context (positive vs. negative stimuli) during the regulation process.

## 4 Discussion

In the present study, we analyzed a dataset of evoked positive and negative empathy responses to explore the bottom-up perception and top-down regulation processes of empathy. We identified brain regions associated with the early (i.e., bilateral IFGtri) and late (i.e., bilateral STS) perception process and the regulation process (i.e., the left dlSFG, the mPFC, and the precuneus). For the perception process, the DCM#1 analysis revealed that excitatory effects were observed from bilateral IFGtri to bilateral STS, while inhibitory effects were observed from bilateral STS to bilateral IFGtri. For the regulation process, the DCM#2 analysis revealed an inhibitory modulation from the dlSFG to the mPFC and from the mPFC to the precuneus in response to positive stimuli, while the inhibitory modulation was only observed from the mPFC to the precuneus in response to negative stimuli. In summary, our study provides direct evidence for a hierarchical neural network organization that underpins the bottom-up perception and top-down regulation processes, thus offering support for contemporary theoretical models of empathy (Decety & Meyer, 2008; Heyes, 2018; Preston & de Waal, 2002). Moreover, our results revealed both shared and valence-specific mechanisms for positive and negative empathy, providing insights that may help extend current models. Notably, the TPJ—commonly regarded as critical for self-other awareness—did not emerge as a common region for positive and negative empathy, suggesting that this process could also be influenced by the valence of the others’ emotions. The results may also have important implications for developing targeted interventions to ameliorate empathy deficits. Impaired emotion recognition has been widely reported among individuals with low empathy, including male domestic violence offenders and those with high levels of psychopathic traits (Kyranides et al., 2022; Nyline et al., 2018). In this context, neuromodulation techniques, such as transcranial magnetic stimulation and transcranial direct current stimulation (Li et al., 2023; Ma et al., 2024), offer promising avenues for modulating the neural circuits underlying empathic processing.

For the early perception process, our GLM analysis revealed significant activations in both bilateral IFGtri and the OFCpost across all three conditions (positive, negative, and neutral). The OFCpost, located in the lateral OFC, has been shown to exhibit a face-specific response to attractiveness in both humans and monkeys (Pegors et al., 2015; Tsao et al., 2008), suggesting that the perception process may be automatically influenced by the attractiveness of the protagonists’ faces. However, the IFGtri exhibited stronger activations than the OFCpost in the current study, and its activation negatively correlated with the protagonists’ positive and negative emotions. Additionally, previous studies have shown that damage to the right IFG impairs the ability to recognize emotional expressions, particularly those involving the eyes (Shamay-Tsoory et al., 2009). Lesion-symptom-mapping studies have also reported that lesions in bilateral frontal and temporal cortices, including the IFG and STS, impair the understanding of others’ facial expressions (Adolphs et al., 2000; Dal Monte et al., 2013). Given that the IFGtri is more closely linked to facial expression recognition than the OFCpost, and its intrinsic connection with the STS (Suchan et al., 2014), we focused solely on the IFGtri in our DCM#1 analysis to prevent the model from becoming overly complex. However, the OFCpost may be included in future studies that aim to assess the influence of facial attractiveness on emotion perception.

DCM#1 results revealed excitatory effects on between-region connections from bilateral IFGtri to bilateral STS and inhibitory effects on connections from bilateral STS to bilateral IFGtri. In other words, the activity in bilateral IFGtri increased the rate of changes in bilateral STS, while the activity in bilateral STS inhibited the rate of changes in bilateral IFGtri. This finding suggests information flows from bilateral IFGtri to bilateral STS, supporting the bottom-up perception process of empathy. Furthermore, the right IFGtri exhibited a positive self-connection parameter in the DCM, suggesting that it responded less to inputs from the network. While the left IFGtri was inhibited by bilateral STS, the right IFGtri was inhibited only by the left STS in response to emotional stimuli. Additionally, GLM#1 revealed stronger activations in the right IFGtri than the left IFGtri (left: *t* = 6.70, right: *t* = 9.87, Supplementary Table S1), a phenomenon also observed in action observation (Molnar-Szakacs et al., 2005). These results may suggest that the right IFGtri plays a more independent role in recognizing others’ facial expressions (i.e., focusing more on external information gathering), while the left IFGtri is more involved in communication with other empathy-related brain regions (i.e., focusing more on internal communication). Although the human brain is anatomically symmetrical, with mirror-like brain regions in the left and right hemispheres, functional asymmetries are prevalent (Wang et al., 2023). This is also true for the IFGtri, as the left IFGtri, part of Broca’s area, plays a more important role in language functions and less in action observation (Konstantopoulos & Giakoumettis, 2023; Qi et al., 2019) and facial expression recognition (Uono et al., 2017) compared with the right IFGtri. Moreover, bilateral IFGtri activations were negatively correlated with participants’ trial-by-trial ratings of others’ emotions, suggesting that clearer recognition of others’ emotions required less engagement of regions associated with the mirror neuron system, and vice versa. This finding complements our main GLM and DCM results: whereas GLM/DCM analyses treated emotional stimuli as a whole, the parametric modulation analysis captured trial-to-trial variations within each category (i.e., positive and negative stimuli), suggesting that the IFGtri is engaged both at the categorical level (GLM/DCM) and at the parametric level of subjective experience, underscoring the complementary relationship between neural activities and behavioral aspects of empathy.

Three brain regions, i.e., the left dlSFG, mPFC, and precuneus, exhibited increased activations in both the positive and negative conditions relative to the neutral condition. These regions were therefore included in the DCM#2 analysis to examine the regulation process. Among these regions, the dlSFG, a part of the DLPFC, is associated with high-level cognitive control over a range of brain regions (Seminowicz & Moayedi, 2017). Lesions in the DLPFC lead to empathy deficits (Hillis, 2014). Therefore, the dlSFG could be considered the top controller in the DCM#2 analysis. The results show that the modulatory effect was inhibitory on the between-region connection from the dlSFG to the mPFC in response to positive stimuli, but not to negative stimuli. The dlSFG has been found to be specifically involved in overriding prepotent emotional biases and social decision making (Rilling & Sanfey, 2009). It has been hypothesized that difficult social decisions often involve competition between emotional processing and higher-level controlled processes, which bias decision making in opposite directions (Rilling & Sanfey, 2009). Whereas emotional processes are driven by subcortical, limbic, and paralimbic structures, higher-level controlled processes rely on the anterior and dorsolateral regions of the prefrontal cortex, as well as areas of the posterior parietal cortex (Rilling & Sanfey, 2009). Our DCM#2 analysis demonstrates that this competition was more pronounced in response to positive stimuli, but not to negative stimuli. Indeed, while positive empathy often fosters a desire to celebrate and enhance others’ well-being, it can also evoke contrasting emotions, such as envy, which is commonly observed in real-life contexts (Ganegoda & Bordia, 2019; Ramachandran & Jalal, 2017). In contrast, negative empathy, which entails understanding and sharing in others’ distress, typically elicits more consistent emotional responses, resulting in helping behaviors that are important for social interaction (Motomura et al., 2015; Salles et al., 2024). Our results suggest that less higher-level control was involved in negative empathy compared with positive empathy, providing further neural evidence for the difference between positive and negative empathy.

Top-down inhibitory modulation was also observed, from the mPFC to the precuneus in response to both positive and negative stimuli. The mPFC plays an important role in numerous cognitive processes, such as emotional regulation, sociability, and empathy, and is notably highly interconnected with subcortical regions, for example, the thalamus, amygdala, and hippocampus (Miller & Cohen, 2001; Xu et al., 2019). The mPFC is also crucial for exerting top-down executive control across various cognitive functions (Engel & Gunnar, 2020; Jobson et al., 2021; Ochsner & Gross, 2005). Therefore, it is possible that the mPFC receives extensive projections from limbic regions and, in turn, inhibits the activity in the precuneus, which is involved in self-referential processing, regardless of whether the emotion is positive or negative. Furthermore, DCM#2 results show that the dlSFG, mPFC, and precuneus were all driving inputs in response to positive stimuli, while only the dlSFG and precuneus were involved in responses to negative stimuli. This observation may suggest that these brain regions work partly in parallel but are intertwined via between-region effective connectivity, especially for positive empathy. However, the current study focused solely on shared ROIs involved in the regulation process of positive and negative empathy, without considering some specific influential factors (e.g., the subject’s sex or the relationship between the subject and the object). Future studies could also explore how these factors modulate top-down regulation effects in valence-specific ROIs.

In conclusion, our study utilized a dataset specifically designed to explore the bottom-up perception and top-down regulation processes of positive and negative empathy. Our findings provide valuable insights into the neural network mechanisms involved in processing positive and negative empathy, supporting theoretical models of empathy. However, there are several limitations in the current study. First, since the stimuli consisted of facial expressions, our results may not generalize to empathy evoked by observing others’ body movements or hearing their voices, particularly with regard to the initial perceptual processing regions (i.e., bilateral IFGtri). Second, empathy encompasses a variety of subprocesses, with both shared and distinct processing occurring for positive and negative empathy. The present study focused solely on shared processing, while distinct brain regions may be engaged in the processing of positive versus negative empathy, for example, automatic responses. Therefore, our findings cannot be generalized to the unique processing mechanisms associated with either positive or negative empathy. Third, in our experimental design, we only considered the happy and sad emotions to evoke positive and negative empathy, respectively. Other emotions, such as anger and fear, are also relevant to empathic processes and may engage distinct neural dynamics (Riddell et al., 2024). Future research could include a broader range of emotion categories to better capture the diversity of empathic responses. Fourth, only young adults were recruited in this study, and future studies should include more diverse samples across different age groups and cultural backgrounds to assess the robustness and generalizability of our findings. Fifth, previous research has shown that affective responses in positive and negative empathy involve different brain regions (Wu et al., 2025). Our experiment was not designed to target variables specifically associated with positive or negative empathy, limiting our ability to examine effective connectivity within affective networks or the interaction between perception and regulation processes. Future studies could focus on variables associated with positive or negative empathy and explore how these variables influence empathic processing (Mwilambwe-Tshilobo et al., 2023; Wu et al., 2023a, 2023b). Sixth, we employed the DCM approach to test our hypothesis, as it is well suited for assessing directed connectivity based on fMRI data and offers advantages over alternatives such as Granger causality (Friston, 2011). However, DCM has inherent limitations. It requires a priori model specification, which may bias the analysis toward hypothesized structures and overlook alternative network organizations. In addition, since DCM operates on fMRI data, the limited temporal resolution can make it difficult to draw firm conclusions about the timing of early versus late neural processes. Furthermore, although DCM ideally relies on well-defined ROIs, our selection was primarily informed by GLM results and supported by the literature. Future research may benefit from adopting more hypothesis-driven or theory-based strategies for ROI selection. Alternative ROI selections could be considered to address different research questions or target other aspects of empathic processing. Addressing these limitations will help provide a more comprehensive understanding of the hierarchical neural organization of empathy processing.

## Supplementary Material

Supplementary Material

## Data Availability

The data and code that support the findings of this study are available from the corresponding author upon reasonable request.
