# Supplementary material for: Neural network dynamics underlying the bottom-up perception and top-down regulation processes of empathy
Source: Imaging Neurosci (Camb). 2025 Nov 3;3:IMAG.a.972. doi: 10.1162/IMAG.a.972 (PMC12583916; doi:10.1162/IMAG.a.972)
Supplement: Supplementary Material [file IMAG.a.972_supp.pdf]

MRI Acquisition and Preprocessing

The functional magnetic resonance imaging (fMRI) data were obtained using a Gradient Echo pulse sequence (TR [Repetition Time] = 800 ms, TE [Echo Time] = 30 ms, Flip Angle [FA] = 60°, Field of View [FOV] = 216 × 216 mm^2, matrix = 90 × 90, slice thickness = 2.4 mm, hyperband slice = 6, no slice spacing, 60 oblique slices). Note that the first five subjects were scanned with a TR of 750 ms, and the remaining participants were scanned with a TR of 800 ms to prevent reaching the upper limit of the scanner. Fieldmap images were obtained before task fMRI images (TE short = 4.9 ms, TE long = 7.4 ms). High-resolution T1-weighted structural images were obtained using an MP-RAGE pulse sequence (FA = 7°, FOV = 256 × 256 mm^2, matrix = 320 × 320, slice thickness = 0.8 mm, 240 sagittal slices).

To preprocess the fMRI data, structural images were firstly corrected for intensity nonuniformity using ANTS 2.5.1. Next, the first 12 fMRI volumes from each functional run were removed to ensure the scanner signal stability. The remaining fMRI data were then preprocessed using SPM 12 toolbox (Statistical Parametric Mapping, http://www.fil.ion.ucl.ac.uk/spm). Specifically, voxel-displacement maps (VDMs) were computed from fieldmap images for each run, and fMRI images were realigned and unwarped using the VDM files as the phase maps while registering to the mean task fMRI image. The bias-corrected structural image was coregistered to the mean task fMRI image and segmented into gray matter, white matter, and cerebrospinal fluid. Subsequently, fMRI images were normalized to the Montreal Neurological Institute space using the deformation field obtained during structural image segmentation, resampled to 2 × 2 × 2-mm^3 isotropic voxels, and smoothed with a 4-mm full-width half-maximum Gaussian kernel.

Table S1

Conjunction Analysis of Brain Regions Significantly Activated by Positive, Neutral, and Negative Stimuli

Cluster size	Hemisphere	Brain regions
9883	M, L, R	Lingual gyrus extending to bilateral fusiform gyrus, IOG, parahippocampal gyrus, right hippocampal gyrus, and right amygdala
1519	L	Middle temporal gyrus, superior temporal gyrus, and middle occipital gyrus
620	R	Triangular part of IFG
524	L	STS
475	R	STS
289	L	Triangular part of IFG
94	L	Posterior part of OFC
63	R	Posterior part of OFC

Note. M: Medial, L: left, R: right; IOG: inferior occipital gyrus; IFG: inferior frontal gyrus; STS: superior temporal sulcus; OFC: orbitofrontal cortex. Significance threshold of $p < .001$ at the voxel level and family-wise error (FWE)-corrected $p < .05$ at the cluster level were applied for brain regions activated for positive, neutral, and negative stimuli, respectively.

Table S2

Conjunction Analysis of Brain Regions for Positive Versus Neutral and Negative Versus Neutral Contrasts

Cluster size	Cluster *p* (FWE)	Hemisphere	Brain regions	*t* value	Peak MNI coordinates		
					X	Y	Z
737	< .001	M	precuneus	8.24	-2	-58	22
2,929	< .001	L	STS	7.73	-56	2	-14
1,449	< .001	R	STS	6.26	56	-62	18
324	< .001	M	mPFC	4.81	-6	48	6
78	.011	L	dlSFG	4.76	-20	32	36

Note. M: Medial, L: left, R: right; STS: superior temporal sulcus; mPFC: medial prefrontal cortex; dlSFG: dorsolateral superior frontal gyrus. Significance threshold of $p < .001$ at the voxel level and family-wise error (FWE)-corrected $p < .05$ at the cluster level were applied.
